# *Sporothrix schenckii* Immunization, but Not Infection, Induces Protective Th17 Responses Mediated by Circulating Memory CD4^+^ T Cells

**DOI:** 10.3389/fmicb.2018.01275

**Published:** 2018-06-12

**Authors:** Alberto García-Lozano, Conchita Toriello, Laura Antonio-Herrera, Laura C. Bonifaz

**Affiliations:** ^1^Unidad de Investigación Médica en Inmunoquímica, Hospital de Especialidades, Centro Médico Nacional Siglo XXI, Instituto Mexicano del Seguro Social, Mexico City, Mexico; ^2^Facultad de Medicina, Universidad Nacional Autónoma de México, Mexico City, Mexico; ^3^Laboratorio de Micología Básica, Departamento de Microbiología y Parasitología, Facultad de Medicina, Universidad Nacional Autónoma de México, Mexico City, Mexico

**Keywords:** *Sporothrix schenckii*, CD4 T cell memory development and survival, skin immunization, cholera toxin, Th1/Th17 immunity

## Abstract

Sporotrichosis is a chronic subcutaneous mycosis caused by the *Sporothrix schenckii* species complex and it is considered an emerging opportunistic infection in countries with tropical and subtropical climates. The host’s immune response has a main role in the development of this disease. However, it is unknown the features of the memory cellular immune response that could protect against the infection. Our results show that i.d. immunization in the ears of mice with inactivated *S. schenckii* conidia (iC) combined with the cholera toxin (CT) induces a cellular immune response mediated by circulating memory CD4^+^ T cells, which mainly produce interleukin 17 (IL-17). These cells mediate a strong delayed-type hypersensitivity (DTH) reaction. Systemic and local protection against *S. schenckii* was mediated by circulating CD4^+^ T cells. In contrast, the infection induces a potent immune response in the skin mediated by CD4^+^ T cells, which have an effector phenotype that preferentially produce interferon gamma (IFN-γ) and mediate a transitory DTH reaction. Our findings prove the potential value of the CT as a potent skin adjuvant when combined with fungal antigens, and they also have important implications for our better understanding of the differences between the memory immune response induced by the skin immunization and those induced by the infection; this knowledge enhances our understanding of how a protective immune response against a *S. schenckii* infection is developed.

## Introduction

Sporotrichosis is a chronic subcutaneous mycosis caused by the *S. schenckii* species complex (Marimon et al., 2007, 2008; López-Romero et al., 2011; Vásquez-del-Mercado et al., 2012). The disease begins with a traumatic lesion in the skin caused by conidia contaminated material, the infective form of *S. schenckii* (Reed et al., 1993; Kauffman, 1999; Rivitti and Aoki, 1999; Ramos-e-Silva et al., 2007; Bonifaz and Vázquez-González, 2010). Immunocompetent individuals usually develop localized cutaneous forms of the infection, while immunocompromised patients generally develop disseminated and systemic forms (Shaw et al., 1989; Heller and Fuhrer, 1991; Gori et al., 1997; Rocha et al., 2001; Carvalho et al., 2002; da Rosa et al., 2005; Silva-Vergara et al., 2005; Carlos et al., 2009). It has been shown that cellular responses mediated by different populations of the innate and adaptive immune response are generated during the *S. schenckii* infection (Miyaji and Nishimura, 1982; Tachibana et al., 1999; Koga et al., 2001; Koga et al., 2002; Martínez-Álvarez et al., 2014). Therefore, subjects with cellular immune deficiencies are more susceptible to systemic infection (Plouffe et al., 1979; Shiraishi et al., 1979, 1992; Dickerson et al., 1983). Recently, some reports have shown that DCs are able to recognize different cell-wall antigens of *S. schenckii* that generate a differential activation of DCs correlating to the development of the Th1/Th17 CD4^+^ T cells response *in vitro* (Uenotsuchi et al., 2006; Verdan et al., 2012; Kusuhara et al., 2014). These cells, developed during the *S. schenckii* infection are important for fungal control and for optimal fungal clearance (Fujimura et al., 1996; Tachibana et al., 1999; Maia et al., 2006; Ferreira et al., 2015). Furthermore, reports have mentioned that the infection could induce a cellular memory immune response because patients with sporotrichosis develop DTH reactions after the i.d. inoculation with sporotrichin (a glycoprotein extract from *S. schenckii*) (Bonifaz and Vázquez-González, 2010). Nowadays, many subpopulations of memory T cells have been described; however, their role in the induction of a protective immune response is still a matter of debate (Antia et al., 2005; Zens and Farber, 2015). Some results show that tissue-resident memory T (T_RM_) cells, a sub-population of effector memory T (T_EM_) cells with an immediate effector function at the site of antigen access, are essential mediators of DTH reactions, and they could elicit this response without participation of the central memory T (T_CM_) cells (Matheu et al., 2008). T_CM_ cells are a population with the capacity to produce high levels of IL-2 and to recirculate from lymph nodes to tissues through lymphatic vessels after activation (Kaech and Wherry, 2007). Other reports mention that T_RM_ cells can protect against the influenza virus infection (Hogan et al., 2001; Teijaro et al., 2011; Zens and Farber, 2015). Furthermore, it has also been shown by still other reports that the control of the *Chlamydia trachomatis* infection requires the participation of both seeding T_RM_ cells and subsequent recruitment of circulating memory T cells (Matheu et al., 2008; Stary et al., 2015). Therefore, there is controversy over the role of distinct memory T cell subpopulations against infection, often depending on the model used for their study. However, participation of different memory T cell subpopulations in fungal infections has only been studied in the *Candida albicans* infection (Park et al., 2017), and there is no experimental evidence that demonstrates the development and participation of different subpopulations of memory T cells during sporotrichosis and other chronical fungal infections.

Previous work in our laboratory showed that i.d. immunization with the CT combined with HEL induces a cellular memory immune response mediated by CD4^+^ T cells with a Th1/Th17 phenotype (Meza-Sánchez et al., 2011). This result indicates that the CT can induce an efficient CD4^+^ T cell response to a co-administered model antigen. However, it is unknown if a similar memory immune response can be elicited by i.d. inoculation of the CT when combined with microbial antigens, such as *S. schenckii* conidia. The aim of this work was to compare the immune responses induced by skin immunization and those induced by infection, by evaluating which memory T cell subpopulations are generated under both conditions. Our results demonstrate that i.d. immunization with the iC combined with the CT induces a cellular immune response —mediated by circulating memory CD4^+^ T cells— that preferentially produces IL-17 and protects mice against a *S. schenckii* infection. In contrast, i.d. inoculation of live conidia (LC) induces an effector phenotype CD4^+^ T cell response that mainly produces IFN-γ. Our findings have important implications for our understanding of the immune response observed during the infection with *S. schenckii*, and they contribute to our knowledge of the different roles T cell memory subsets play in the development of a protective immune response against fungal infections.

## Materials and Methods

### Fungi

In all experiments, *S. schenckii*, strain EH-143, was used. It was obtained from a patient with cutaneous-lymphatic sporotrichosis. The species identity was based on their phenotypic and genotypic characteristics (Mesa-Arango et al., 2002) and it was verified by analyzing calmodulin genes sequences (Madrid et al., 2009). It is part of the Culture Collection of Fungal Pathogens from the Laboratorio de Micología Básica, Departamento de Microbiología y Parasitología, Facultad de Medicina, Universidad Nacional Autónoma de México (UNAM), and it is registered at the World Federal Culture Collection (WFCC) as BMFM-UNAM 834.

According to reported by Uenotsuchi et al. (2006), the fungus was cultured on Sabouraud’s agar for 7 days at 28°C to promote the growth of the mycelial form, which consist of hyphae and conidia. Fungal cultures were suspended in PBS and heat-inactivated by a 2-h incubation in a 60°C water bath. The inactivated fungus was filtered through Whatman paper No. 1 to remove all hyphae and pseudohyphae and to recover only pure conidia.

The iC in the final filtrate were cultured on Sabouraud’s agar at 28°C to confirm their inactivation. Afterward, the iC were counted in a Neubauer chamber and the conidia form was confirmed. Finally, conidia were used in a final concentration of 5 × 10^4^ conidia/μL and stored at 4°C until use.

### Mice

C57BL/6 mice were provided by the Experimental Medicine Unit of UNAM. All animal experiments were performed in 8 to 12-week-old mice in accordance with the Institutional Ethics Committee. (Comite local de investigación en salud) Protocol number 3601. All procedures for animals were approved by the Animal Ethics Committee of the Faculty of Medicine at the UNAM, and followed the Mexican Official Guide (NOM 062-ZOO-1999) for the care and use of laboratory animals. The animals were free of parasites or pathogens, and females were nulliparous and non-pregnant. Animals were fed LabDiet 5010 Autoclavable Rodent diet (LabDiet, United States) and water *ad libitum.*

### Cytokine Secretion Determination

C57BL/6 mice were i.d. injected in the ears with PBS, iC (5 × 10^5^ conidia), the CT (1 μg), iC in combination with the CT or LC (5 × 10^5^ LC). After 14 days, mice were sacrificed and 3 × 10^5^ cells were obtained from the sdLN of the ears. These cells were suspended in 100 μl of supplemented RPMI 1640 medium (Biowest, France) and they were re-stimulated with 10 μl of PBS or 5 × 10^5^ iC. After 24 h, cell free culture supernatants were assessed for the presence of cytokines using the Mouse Th1/Th2/Th17 Cytometric Bead Array Kit (BD Biosciences, United States), according to the manufacturer’s instructions, and they were analyzed using flow cytometry.

### Identification of Stained Conidia of *S. schenckii* After Intradermal Inoculation

The transgenic mice C57BL/6 that express the green fluorescent (GFP) under the major histocompatibility complex class II molecule (MHC-II) promoter were i.d. injected in the ears with PBS, iC (1 × 10^6^ iC) stained with the CellMask Orange Plasma Membrane Stain (Thermo Fisher Scientific, United States), or LC (1 × 10^6^ LC) stained with CellMask Orange Plasma Membrane Stain. Six hours later the ears were removed and treated with 0.5 M EDTA for 2 h and then were washed with PBS for 20 min. The epidermal layer was then separated from the dermal layer, washed, and acetone-fixed for 20 min at -20°C. Afterward, the epidermal sheets were mounted with VectaShield (Vector Laboratories, Burlingame, CA, United States) and sealed. The images were obtained with a Leica TCS SP8x Confocal Microscope (Wetzlar, Germany) and analyzed with Leica Application Suite Advanced Fluorescence Lite software (Leica Microsystems, Mannheim, Germany).

For the staining, 1 × 10^6^ conidia of *S. schenckii* were incubated with 1000 μL of CellMask Orange 1× for 30 min at 37°C, after that the conidia were washed twice with PBS (PBS).

### Th1/Th17 Phenotype of CD4^+^ T Cells and CD4^+^ T Cell Memory Populations in sdLN

C57BL/6 mice were i.d. injected in the ears with PBS, iC (5 × 10^5^ iC), the CT (1 μg), iC in combination with the CT or LC (5 × 10^5^ LC). After 14 days, mice were sacrificed and 3 × 10^5^ sdLN cells were obtained and co-cultivated with 1 × 10^5^ CD11c^+^ cells (DCs) that were isolated from the sdLNs of naive C57BL/6 mice using anti-mouse CD11c MACS MicroBeads (Miltenyi Biotec, United States). The DCs were plated with 5 × 10^5^ iC for 24 h before being co-cultured with the sdLN cells. DC and 3 × 10^5^ sdLN cells were co-cultured for 7 days. During the last 4 h, a cell stimulation cocktail and the protein transport inhibition cocktail (eBioscience-Affymetrix, Santa Clara, CA, United States) were added to assess the production of cytokines via intracellular staining.

The cells were then stained for surface markers CD45, CD4 and TCR-β and treated with BD Cytofix/Cytoperm (Fixation/Permeabilization Kit) (BD Biosciences, San Diego, CA, United States) for intracellular staining, following the manufacturer’s instructions. The intracellular cytokines were detected using anti-IFN-γ (Clone: XMG1.2, BioLegend, San Diego, CA, United States) and anti-IL-17 (BD Biosciences, San Jose, CA, United States) antibodies.

In addition, 3 × 10^5^ sdLN cells were plated with 5 x 10^5^ iC for 7 days and these cells were stained for T cell memory surface markers (CD45, CD4, TCR-β, CD44, CD62L, CCR7, and CD69) and they were analyzed by flow cytometry.

The totality of the CD45^+^ cells present in the sdLN was calculated using absolute counting beads (CountBright, Life technologies, Eugene, OR, United States) in a FACS Canto Flow cytometer (BD Biosciences, San Jose, CA, United States).

### Cytokine Production and CD4^+^ T Cell Memory Populations in the Skin

C57BL/6 mice were injected i.d. in the ears with iC (5 × 10^5^ iC), the CT (1 μg), iC with the CT or LC (5 × 10^5^ LC). After 31 or 14 days, mice were sacrificed and the ears were obtained. The ears were treated with Liberase TL (Roche, Mannheim, Germany) and DNAase I (Roche Diagnostics, Mannheim, Germany) and after 45 min, they were cut into small pieces and subjected to constant shaking at 200 rpm. The cells obtained from the ears were suspended in 100 μL of supplemented RPMI medium and were plated with 5 × 10^5^ iC overnight; in the last 4 h of co-culture, a cell stimulation cocktail and the protein transport inhibition cocktail (eBioscience-Affymetrix, Santa Clara, CA, United States) were added to assess the production of cytokines via intracellular staining.

The cells were stained for surface markers (CD45, CD4, TCR-β) and treated with BD Cytofix/Cytoperm (Fixation/ Permeabilization Kit) (BD Biosciences, San Diego, CA, United States) for intracellular staining, following the manufacturer’s instructions. The intracellular cytokines were detected using anti-IFN-γ, anti-IL-17 and anti-TNF-α antibodies.

Also, all skin cells of immunized mice were plated with 5 × 10^5^ iC for 7 days and then, they were stained for memory surface markers (CD45, CD4, TCR-β, CD44, CD62L, CCR7, and CD69) and analyzed by flow cytometry.

The total number of the CD45^+^ cells present in the skin was calculated using absolute counting beads (CountBright, Life technologies, Eugene, OR, United States) in the FACS Canto Flow cytometer.

### DTH Response

C57BL/6 mice were i.d. injected in the ears with PBS (10 μL), iC (5 × 10^5^ iC), the CT (1 μg), iC with the CT (1 μg) or LC (5 × 10^5^ LC). After 31 days, ear thickness was measured and mice were challenged with i.d. iC in the ears (5 × 10^5^ iC). Ear thickness was measured 24 and 48 h post-challenge.

### CD4^+^ T Cell Depletion and Staining of CD4^+^ T Cells in sdLN and Skin

C57BL/6 mice were i.d. injected in the ear with iC (5 × 10^5^ iC) plus the CT (1 μg). After 31 days, ear thickness was measured and mice were intraperitoneally (i.p.) inoculated with anti-CD4 monoclonal antibody (clone GK 1.5) (250 μg) or irrelevant antibody (Rat IgG1 κ Iso Control, Clone eBRG1, eBioscience) (250 μg). After 24 h of inoculation with the anti-CD4 or irrelevant antibody, ear thickness was measured and mice were challenged with an i.d. injection in the ears with iC (5 × 10^5^ iC). Ear thickness was measured 24 and 48 h post-challenge. The sdLN cells and ear skin cells were obtained, stained for CD45, CD4, TCR-β, CD44, CD62L, CCR7, and CD69 markers, and analyzed by flow cytometry.

The total number of the CD45^+^ cells, present in the sdLN and in the skin, was calculated using absolute counting beads (CountBright, Life technologies, Eugene, OR, United States) in the FACS Canto Flow cytometer.

### Infection Model

C57BL/6 mice were i.d. injected in the ears with two different amounts of LC (5 × 10^5^ and 10 × 10^6^ LC). After 17, 24 and 31 days, the area of the local lesions in the ears was calculated with the ImageJ Software (ImageJ Software, National Institutes of Health) and the appearance of tail granulomas was evaluated up until the 60th day post-inoculation.

### Protection Model

C57BL/6 mice were i.d. injected in the ears with PBS (10 μL), iC (5 × 10^5^ iC), the CT (1 μg) or iC plus the CT (iC+CT). After 31 days of immunization, mice were challenged with an i.d. injection in the ears of 10 × 10^6^ LC. After 17 and 24 days post-infection, the area of the local lesions was calculated with the Image J software and the appearance of granulomas in the tail of infected mice was evaluated up until the 75th day post-infection. Furthermore, some mice who were immunized with iC+CT were i.p. injected with anti-CD4 monoclonal antibody (250 μg) or irrelevant antibody (250 μg) 24 h before immunization or 24 h before the challenge and before the skin lesion area was calculated.

### Flow Cytometry

The total number of the CD45^+^ cells, present in the sdLN and in the skin, was calculated using absolute counting beads (CountBright, Life technologies, Eugene, OR, United States) in the FACS Canto Flow cytometer.

The cells that were stained for extracellular markers or intracellular cytokines were analyzed using a FACS Canto flow cytometer (BD Biosciences, San Jose, CA, United States). The results were analyzed using FlowJo (Tree Star, Ashland, OR, United States) software.

### Statistical Analysis

Statistical analysis was performed using GraphPad Prism 5.0 (La Jolla, CA, United States) software. One-way ANOVA or two-way ANOVA and parametric Bonferroni post-test were used to calculate the statistical significance between the groups. All *p-*values less than 0.05 were considered as statistically significant.

## Results

### Intradermal Inoculation of iC of *S. schenckii* in Combination With the CT Induces IFN-γ, IL-17 and a Higher Expression of IL-2, in Contrast to Skin Infection That Highly Express TNFα

**FIGURE 1:**
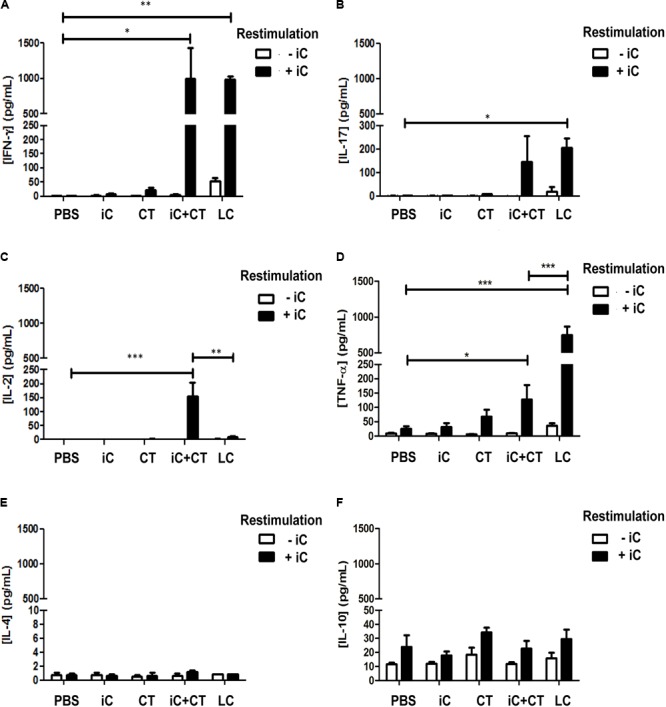
Intradermal (i.d) inoculation of inactivated *S. schenckii* conidia (iC) in combination with the CT induces IFN-γ, IL-17 and a higher expression of IL-2, in contrast to skin infection that highly express TNFα. C57BL/6 mice were injected i.d. in the ears with either PBS, iC (5 × 10^5^ iC), the CT (1 μg), iC plus the CT (iC+CT) or live conidia (LC) (5 × 10^5^ LC). After 14 days, all the sdLN cells were obtained and all 3 × 10^5^ sdLN cells were re-stimulated (+iC) or not (–iC) with 5 × 10^5^ iC. After 24 h of re-stimulation, cell-free culture supernatants were assessed for the presence of IFN-γ **(A)**, IL-17 **(B)**, IL-2 **(C)**, TNF-α **(D)**, IL-4 **(E)** and IL-10 **(F)**, using the Mouse Th1/Th2/Th17 Cytometric Bead Array Kit (BD Biosciences) and flow cytometry. All graphs show mean ± standard error of the mean (SEM). Data were obtained from three independent experiments with three mice per group. Two-way ANOVA and Bonferroni post-test were performed; ^∗^*p* < 0.05; ^∗∗^*p* < 0.01; ^∗∗∗^*p* < 0.001.

It has been previously reported that i.d. immunization in the ear with HEL in combination with the CT induces a Th1/Th17 response (Meza-Sánchez et al., 2011); therefore, to evaluate if a similar immune response could be elicited by the i.d. inoculation in the ear with the CT in combination with microbial components, mice were inoculated in the ear with either heat inactivated *S. shenckii* conidia (iC), in the presence or absence of the CT, or with LC. Initially, cytokine expression was evaluated in the sdLN. The totality of sdLN cells from different groups of mice were co-cultured with DCs loaded with iC. The sdLN cells from mice inoculated with iC+CT or LC expressed INF-γ and IL-17 at similar levels (**Figures 1A,B**). However, the sdLN cells from mice inoculated with iC+CT expressed significantly higher levels of IL-2, compared with the sdLN cells from LC mice (**Figure 1C**). In contrast, sdLN cells from mice infected with LC expressed significantly more TNF-α compared with the sdLN cells from the mice inoculated with iC+CT (**Figure 1D**). The expression of these cytokines was not observed in the sdLN cells from mice inoculated with PBS, iC in the absence of the CT or with the CT alone. The expression of IL-4 was not observed in any of the evaluated groups (**Figure 1E**) and no significant differences were observed in IL-10 expression (**Figure 1F**). To rule out that the differences in cytokine expression observed after inoculation with LC or iC+CT were not caused by a change in the fungus morphology after mice inoculation, the iC or the LC were labeled and inoculated i.d in the ears of mice that express GFP under the MHC-II promoter that allow identification of skin DCs. After 6 h of inoculation, the ears were removed to obtain epidermal sheets. As it can be observed in Supplementary Figure 1 both LC and the iC (red label) are in contact with skin DC (green). Importantly, the average size of 4 μm and the morphology of the LC are very similar compared with the iC, suggesting that the differences in the expression of cytokines are consequence of the infection versus immunization and not due to a change into yeast morphology. All together these results suggest that both the immunization (iC+CT) and the infection (LC) induce a similar INF-γ/ IL-17 response; however, the higher expression of TNFα induced by the infection suggest that this cytokine could be implicated in tissue damage. In contrast the higher expression of IL-2 by the sdLN cells from the mice inoculated with iC+CT suggests that the immunization could elicit a different memory immune response compared to the infection. Remarkably, the results indicate that the CT can induce an INF-γ/IL-17 response after co-administration with related microbial components.

### Inactivated *S. schenckii* Conidia With the CT Induce a Strong Th17 Response, Compared to Skin Infection

**FIGURE 2:**
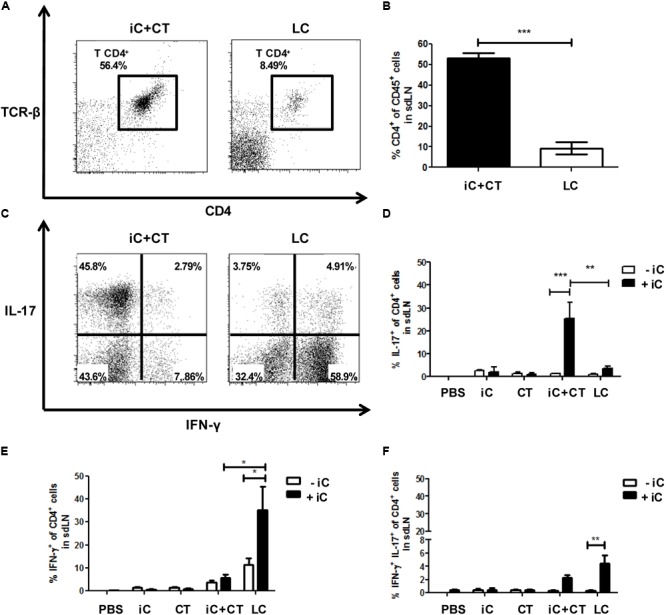
Inactivated conidia of *S. schenckii* in combination with the CT induce a strong Th17 response compared to the skin infection. C57BL/6 mice were i.d. injected in the ears with PBS, iC (5 × 10^5^ iC), the CT (1 μg), iC+CT or LC (5 × 10^5^ LC). After 14 days, 3 × 10^5^ all sdLN cells were obtained and co-cultured for 7 days with 1 × 10^5^ dendritic cells (DCs) loaded (+iC) or not (–iC) with iC (5 × 10^5^ iC). After 7 days of co-culture, cells were stained for the surface markers CD45, CD4, and TCR-β and for the intracellular cytokines IFN-γ and IL-17. **(A,C)** Representative plots show the frequency of populations CD45^+^CD4^+^TCR- β^+^ **(A)** and the expression of the cytokines IFN-γ and IL-17 in the CD4^+^ T cells **(C)**. **(B)** The frequency of populations CD45^+^CD4^+^TCR-β^+^ was obtained. **(D–F)** The frequencies of populations IL-17^+^ **(C)** IFN-γ^+^ **(D)** and IL-17^+^IFN-γ^+^ **(E)** from CD4^+^ T cells were also obtained. All graphs show mean ± SEM data from two independent experiments with three mice per group. One-way ANOVA **(B)** or two-way ANOVA **(D–F)** and Bonferroni post-test were performed; ^∗^*p* < 0.05; ^∗∗^*p* < 0.01; ^∗∗∗^*p* < 0.001.

To determine the *S. schenckii* conidia-specific CD4^+^ T cells and to evaluate their expression of cytokines; DCs loaded with iC were co-cultured for 7 days with all sdLN cells from mice immunized with iC+CT or LC, and CD4^+^ T cell expansion and intracellular cytokine expression were evaluated by flow cytometry in CD4^+^ T cells. Unexpectedly, there was a significant increase in the frequency of CD4^+^ T cells obtained from the mice immunized with iC+CT compared with the mice inoculated with LC (**Figures 2A,B**). In addition, the frequency of IL-17^+^ CD4^+^ T cells was significantly higher in the mice immunized with iC+CT compared with the mice immunized with LC (**Figures 2C,D**). In contrast, the frequency of IFN-γ^+^ CD4^+^ T cells was higher in the mice inoculated with LC, compared with the iC+CT immunized mice (**Figure 2E**). Also, the frequency of IL-17^+^/INF-γ^+^ CD4^+^ T cells was similar in the mice immunized with iC+CT or LC (**Figure 2F**), as opposed to the very few CD4^+^ T cells expressing these cytokines, which were detected in mice inoculated with PBS, iC or the CT. These results indicate that the immunization with iC in combination with the CT can induce a predominant Th17 response, whereas *S. schenckii* skin infection predominantly induces an IFN-γ response.

### Inactivated Conidia of *S. schenckii* in Combination With the CT Induce a Response of CD4^+^ T Cells With a Central and an Effector Memory Phenotype, Compared to the Skin Infection

Considering the increase in the frequency of CD4^+^ T cells and the high production of IL-2 obtained from the mice immunized with iC+CT, compared with the mice inoculated with LC, next we evaluated the memory phenotype of CD4^+^ T cells present in the sdLN. All cells present in the sdLN from mice immunized with iC+CT or LC were co-cultured for 7 days with iC, and the expression of surface memory markers was evaluated by flow cytometry in CD4^+^ T cells.

**FIGURE 3:**
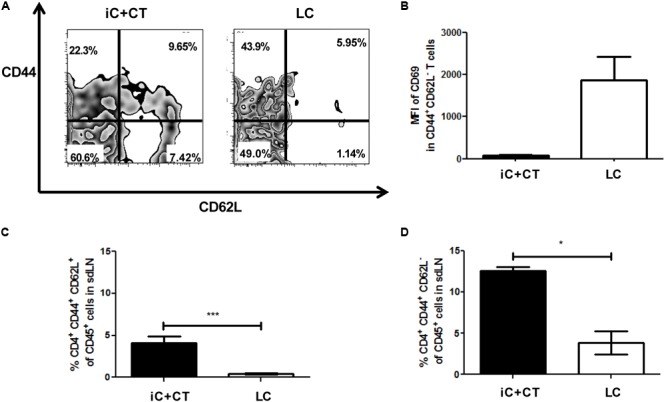
Inactivated conidia of *S. schenckii* in combination with the CT induce a response of CD4^+^ T cells with central (T_CM_) and effector (T_EM_) memory phenotypes, compared to the skin infection. C57BL/6 mice were i.d. injected in the ears with iC+CT or LC (5 × 10^5^ LC). After 14 days, all 3 × 10^5^ sdLN cells were obtained and co-cultivated during 7 days with 5 × 10^5^ iC. After 7 days of co-cultivation, these cells were stained for the surface markers CD45, CD4, TCR-β, CD44, CD62L and CD69. **(A,B)** A representative plot shows the frequencies of populations CD44^+^ CD62L^+^ and CD44^+^CD62L^-^ from CD4^+^ T cells **(A)**; also, the MFI of CD69 in these populations was calculated **(B)**. **(C,D)** The frequency of populations CD4^+^CD44^+^CD62L^+^ (T_CM_ phenotype) **(C)** and CD4^+^CD44^+^CD62L^-^ (T_EM_ phenotype) **(D)** from CD45^+^ cells was obtained. All graphs show mean ± SEM data from two independent experiments with three mice per group. Statistical analysis was performed with one-way ANOVA and Bonferroni post-test; ^∗^*p* < 0.05; ^∗∗∗^*p* < 0.001.

Remarkably, in the mice immunized with iC+CT, there is a CD4^+^ T cell population expressing CD62L and CD44, which is significantly higher in these mice (**Figures 3A,C**). Moreover, there is a population expressing CD44^+^ and CD62L^-^ that is also higher in the mice immunized with iC+CT (**Figures 3A,D**). The expression of these memory markers in both populations and the low expression of CD69 in the population with CD44^+^ and CD62L^-^ markers (**Figure 3B**) suggest that they could correspond to central memory and effector memory, respectively. In contrast, in the mice infected with LC, the population is nearly non-existent with CD44^+^ and CD62L^-^ expressing marker CD69, which could correspond to effector CD4^+^ T cells (**Figures 3A,B,D**). Altogether, these results indicate that the immunization with iC in combination with the CT can induce a CD4^+^ T cell response with a central and an effector memory phenotype in the sdLN, whereas the response induced by a *S. schenckii* skin infection has an effector phenotype in the absence of CD4^+^ T cells with a classic memory phenotype.

### An Infection With *S. schenckii* Induces a Strong Th1 and Th17 Response in the Skin

**FIGURE 4:**
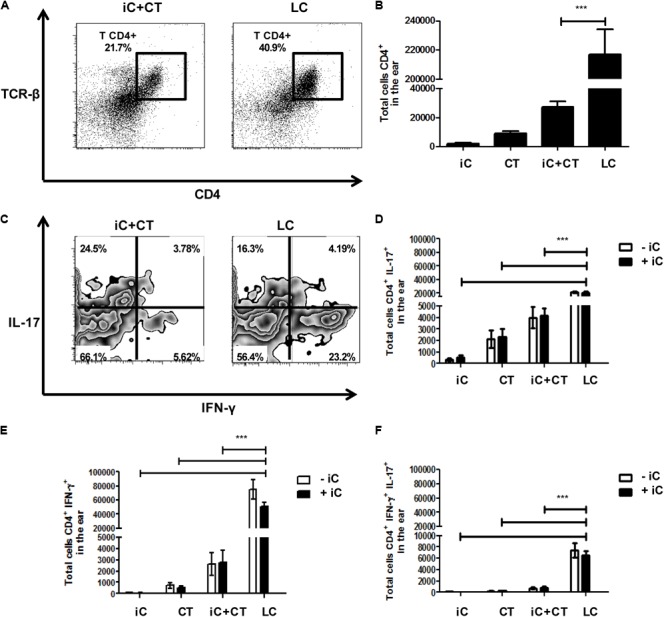
An infection with *S. schenckii* induces a strong INF-γ/ IL-17 response in the skin. C57BL/6 mice were i.d. injected in the ears with iC (5 × 10^5^ iC), the CT (1 μg), iC+CT or LC (5 × 10^5^ LC). After 31 days, all ear skin cells were obtained and re-stimulated (+iC) or not (–iC) with 5 × 10^5^ iC overnight, and finally, these cells were stained for the surface markers CD45, CD4 and TCR-β and for the intracellular cytokines IFN-γ and IL-17. **(A,C)** Representative plots show the frequency of populations CD45^+^CD4^+^TCR- β^+^ **(A)** and the expression of the cytokines IFN-γ and IL-17 in the CD4^+^ T cells after Iono/PMA re-stimulation **(C)**. **(B)** The total numbers of CD45^+^CD4^+^TCR-β^+^ cells were obtained. **(D–F)** The total numbers of IL-17^+^ **(C)**, IFN-γ^+^ **(D)** and IL-17^+^IFN-γ^+^ **(E)** from CD4^+^ T cells were also obtained. All graphs show mean ± SEM data from two independent experiments with three mice per group. Statistical analysis was performed with one-way ANOVA **(B)** or two-way ANOVA **(D–F)** and Bonferroni post-test; ^∗^*p* < 0.05; ^∗∗^*p* < 0.01; ^∗∗∗^*p* < 0.001.

In view of the differences observed in the phenotype of CD4^+^ T cells present in sdLN after inoculation with iC+CT, compared to inoculation with LC, we next evaluated the presence of CD4^+^ T cells and the intracellular cytokine expression in the inoculation site. We observed an increase in the frequency and in the total numbers of CD4^+^ T cells in the skin of the mice infected with LC, compared with the iC+CT immunized mice (**Figures 4A,B**). Importantly, the frequency and the total number of IFNγ^+^ CD4^+^ T cells are significantly higher in the mice infected with LC, compared with the mice inoculated with iC+CT (**Figures 4C,E**). After Iono/PMA re-stimulation, the frequency of IL-17^+^ CD4^+^ T cells is similar in mice inoculated with iC+CT and in mice inoculated with LC (**Figure 4C**), but the total number of IL-17^+^ CD4^+^ T cells is higher in mice inoculated with LC (**Figure 4D**). In addition, the frequency of IFNγ^+^/IL-17^+^ CD4^+^ T cells is similar in mice inoculated with iC+CT and with LC (**Figure 4C**), although the number is higher in infected mice (**Figure 4F**). No differences were observed by the presence of the iC during re-stimulation. In addition, the frequency and total numbers of TNFα^+^ CD4^+^ T cells is higher in the mice infected with the LC compared with the mice inoculated with the iC+CT (Supplementary Figure 2). These results suggest that the infection with *S. schenckii* induces a strong Th1 (IFN-γ and TNFα) and Th17 response in the skin.

### The CT in Combination With *S. schenckii* Conidia Induce a Superior DTH Reaction, Compared to the Infection, Which Is Dependent on Circulating CD4^+^ T Cells

**FIGURE 5:**
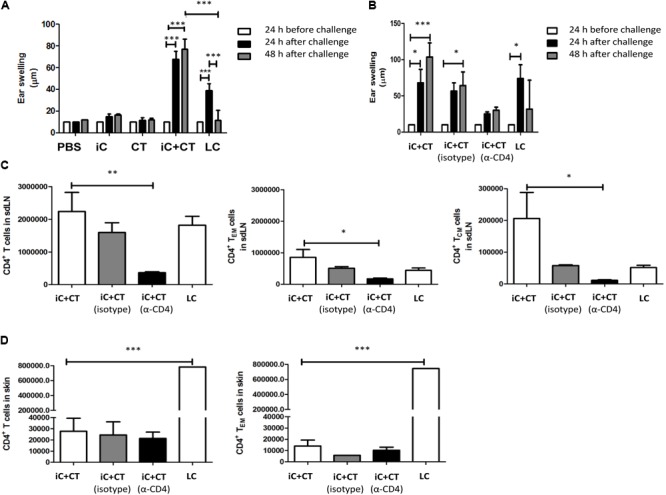
Inactivated conidia of *S. schenckii* in combination with the CT induce a higher delayed-type hypersensitivity (DTH) reaction than the infection, which depends on circulating CD4^+^ T cells. **(A)** C57BL/6 mice were i.d. injected in the ear with PBS (10 μL), iC (5 × 10^5^ iC), the CT (1 μg), iC+CT or LC (5 × 10^5^ LC). After 31 days, ear thickness was measured, and mice were challenged with an i.d. injection in the ears with iC (5 × 10^5^ iC). After 24 and 48 h post-challenge, ear thickness was measured. **(B–D)** C57BL/6 mice were i.d. injected in the ear with iC+CT or LC. After 31 days, mice immunized with iC+CT were intraperitoneally (i.p.) inoculated with PBS, anti-CD4 (α-CD4) monoclonal antibody (250 μg) or irrelevant (isotype) antibody (250 μg), while mice injected with LC were i.p. inoculated with PBS only. After 24 h of inoculation with PBS, anti-CD4 or irrelevant antibody, ear thickness was measured and mice were challenged with the i.d. injection in the ears with iC (5 × 10^5^ iC). Ear thickness was measured 24 and 48 h post-challenge **(B)**. The sdLN cells and ear skin cells were obtained and stained for the markers CD45, CD4, TCR-β, CD44, CD62L, CCR7, and CD69. The total numbers of the CD4^+^ T cells, central memory CD4^+^ T cells (T_CM_) and effector memory CD4^+^ T cells (T_EM_) present in sdLN were calculated **(C)**. The total numbers of the CD4^+^ T cells and effector memory CD4^+^T cells present in the ear skin were calculated **(D)**. All graphs show mean ± SEM data from two independent experiments with three mice per group. Statistical analysis was performed with two-way ANOVA **(A,B)** or one-way ANOVA **(C,D)** and Bonferroni post-test; ^∗^*p* < 0.05; ^∗∗^*p* < 0.01; ^∗∗∗^*p* < 0.001.

In consideration of the cytokine production observed in the skin and the different memory phenotypes of CD4^+^ T cells detected in sdLN, we evaluated the ability of these cells to respond to a challenge *in vivo* with iC. Mice were inoculated with iC+CT or LC, and after 1 month of immunization, they were challenged in the ear with the iC. As can be observed in **Figure 5A**, the inoculation with iC+CT induced a stronger DTH reaction after 24 h of challenge, compared with mice immunized with LC. Importantly, the DTH induced by immunization with the iC+CT is maintained 48 h after iC challenge. In contrast, the DTH induced by LC is less intense and it is only observed 24 h after challenge, as it is not maintained 48 h post-challenge. The DTH response was not observed in mice inoculated with PBS, iC or the CT (**Figure 5A**). Importantly, the DTH response induced by iC+CT was observed after 1 month of immunization, indicating the presence of memory CD4^+^ T cells. Therefore, to evaluate which subpopulations of memory T cells are involved in the development of the DTH response induced by skin immunization with iC+CT, mice were depleted of CD4^+^ T cells by i.p. inoculation with an anti-CD4 (α-CD4) monoclonal antibody 24 h before challenge. As it can be observed in **Figure 5B**, the inoculation of anti-CD4 monoclonal antibody (250 μg) inhibits the development of the DTH response. Furthermore, i.p. administration of anti-CD4 antibody shows a significant reduction in the total number of CD4^+^ T cells, with a central and an effector memory phenotype, present in the sdLN (**Figure 5C**). In contrast, there is no reduction in the totality of CD4^+^ T cells, with an effector memory phenotype, present in the ear (**Figure 5D**). As previously mentioned, there are more T_CM_ and T_EM_ cells present in the sdLN in the mice immunized with iC+CT, compared with the infected mice (**Figure 5C**). However, the total number of CD4^+^ T cells with an effector memory phenotype in the skin of infected mice is higher than the number in immunized mice (**Figure 5D**). These results are similar to those observed in **Figures 3, 4**. Altogether, these results indicate that the inoculation in the ear with *S. schenckii* conidia combined with the CT induces a superior DTH reaction, compared to the infection, which is dependent on circulating memory CD4^+^ T cells.

### Inactivated Conidia of *S. schenckii* in Combination With the CT Protect Against Infection, as Mediated by the Circulating Memory CD4^+^ T Cell Response

**FIGURE 6:**
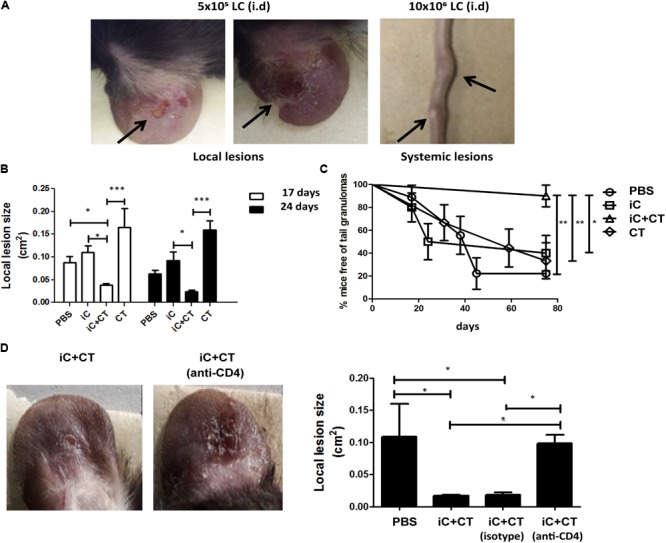
Inactivated conidia of *S. schenckii* combined with the CT induce protection against infection, mediated by the circulating memory CD4^+^ T cell response. **(A)** C57BL/6 mice were injected i.d. in the ears with LC (5 × 10^5^ or 10 × 10^6^). After 17 days, the appearance of local lesions or granulomas in the tail of infected mice was evaluated. **(B–D)** C57BL/6 mice were injected i.d. in the ears with PBS (10 μL), iC (5 × 10^5^ iC), the CT (1 μg) or iC+CT. After 31 days, mice were infected by i.d. injection in the ears with 10 × 10^6^ LC. After 17 and 24 days post-infection, the local lesion area was calculated **(B)** and the appearance of granulomas in the tail of infected mice was evaluated **(C)**. Additionally, the mice immunized with iC+CT were i.p. injected 24 h before the infection with anti-CD4 monoclonal antibody (250 μg) or isotype antibody (250 μg) and the local lesion area of infected mice was calculated **(D)**. The pictures show representative local and systemic lesions in the infected mice. All graphs show mean ± SEM data from three independent experiments with three mice per group. Statistical analysis was performed with one-way ANOVA **(B,D)** and Bonferroni post-test; ^∗^*p* < 0.05; ^∗∗∗^*p* < 0.001.

Considering that immunization with inactivated *S. schenckii* conidia combined with the CT can induce a central memory Th17 response and that the DTH is dependent on circulating memory CD4^+^ T cells, we next evaluated the protection induced by immunization with iC+CT, after a challenge with live infective *S. schenckii* conidia. With this aim in mind, we developed a skin infection model in mice which allowed us to observe local and systemic lesions after i.d. inoculation with different amounts of LC. The results showed that although mice developed skin lesions after 5 × 10^5^ LC i.d. ear inoculation, there are no tail granulomas. In contrast, after 10 × 10^6^ LC i.d. ear inoculation, mice develop both local and systemic lesions (**Figure 6A**). Interestingly, the immunization with iC+CT shows a significant reduction of the skin lesion area after 17 or 24 days of infection, compared with the mice inoculated with PBS, iC or the CT (**Figure 6B**). Importantly, the immunization with iC+CT also shows a significant increase in the percentage of mice, free of tail granulomas (90%), compared with the mice inoculated with PBS, iC or the CT (**Figure 6C**). Finally, to evaluate the role of circulating memory CD4^+^ T cells in protecting against a *S. schenckii* infection, the skin lesion area was evaluated. In **Figure 6D**, a significant reduction in the lesion area of the mice immunized with iC+CT compared with the mice inoculated with PBS can be observed. However, when mice were treated with systemic anti-CD4 monoclonal antibody, to deplete CD4^+^ T cells with circulating memory phenotype present in the sdLN, during the infection (13 days), the mice had a significant increase in the skin lesion area, suggesting an important role for the circulating memory CD4^+^ T cells in the optimal control of a *S. schenckii* infection (**Figure 6D**). These results indicate that i.d. immunization in the ear of iC of *S. schenckii* in combination with the CT induced a local protection mediated by circulating memory CD4^+^ T cells.

## Discussion

In past years, some reports have shown that an adaptive immune response can be generated during a *S. schenckii* infection, and that it is important for the control and clearance of the infection (Miyaji and Nishimura, 1982; Tachibana et al., 1999; Koga et al., 2002; Maia et al., 2006; Uenotsuchi et al., 2006; Verdan et al., 2012; Kusuhara et al., 2014; Ferreira et al., 2015). However, it was unknown if the infection induced a memory cellular immune response. Furthermore, we recently demonstrated in our laboratory that skin immunization with the CT combined with HEL induced a cellular memory immune response and a Th1/ Th17 phenotype (Meza-Sánchez et al., 2011). Nevertheless, it was unknown if the skin immunization with the CT combined with microbial antigens could induce a similar immune response. Here, we report that i.d. immunization in mice’s ears with inactivated *S. schenckii* conidia combined with the CT induced a cellular immune response mediated by CD4^+^ T cells with a circulating memory phenotype. These cells mainly produce IL-17 and they can protect mice against the infection. In contrast, the infection induces a potent immune response in mice’s skin mediated by CD4^+^ T cells with an effector phenotype that produces high levels of IFN-γ and TNFα. Recently, some articles have reported that a Th1/Th17 cellular immune response was important for fungal control and for optimal fungal clearance during a *S. schenckii* infection (Fujimura et al., 1996; Uenotsuchi et al., 2006; Koga et al., 2002; Verdan et al., 2012; Ferreira et al., 2015). However, these results were obtained *in vitro* or in mice infection models using *S. schenckii* yeasts inoculated by different systemic routes and mostly in the BALBc background (Kusuhara et al., 2014; Ferreira et al., 2015), which is a more susceptible strain where a predominant Th1 response has been reported (Alba-Fierro et al., 2016). In contrast, in C57BL/6 background we found a Th1/Th17 immune response using *S. schenckii* conidia, which is the infective structure, and mice were inoculated through the skin, which is the most frequent form of infection (Bonifaz and Vázquez-González, 2010). However, it would be relevant to determine if in our infection or immunization conditions the immune response is similar in different strains of mice. There have been some reports indicating that *S. schenckii* conidia change to yeast after infection (Ramos-e-Silva et al., 2007; Bonifaz and Vázquez-González, 2010; Vásquez-del-Mercado et al., 2012) however, in our model at least at the initial contact with DC in the skin the conidia seem to maintain their morphology. The development of the Th1/Th17 immune response obtained through our skin infection model with conidia is relevant because although conidia and yeasts share molecules, they potentially might generate different immune responses (Fernandes et al., 2000; Uenotsuchi et al., 2006; Guzman-Beltran et al., 2012).

Previous reports have demonstrated that both, iC or yeast, or a fungal extract of these forms were capable of activating DCs *in vitro* (Uenotsuchi et al., 2006; Verdan et al., 2012; Kusuhara et al., 2014). However, our results show that i.d. inoculation with iC alone does not induce a cellular immune response; it is necessary to inoculate it with the CT to induce this response. These results suggest that there are molecules that are necessary for the induction of a cellular immune response, which are present in the LC and lost during heat inactivation. Additionally, our results prove the potential of the CT as a potent skin adjuvant when combined with fungal antigens such as *S. schenckii* conidia.

We also show important differences between the immune response induced by the skin immunization with iC+CT and the response induced by infection. One of the main differences was the differential production of IFN-γ and IL-17. While the infection preferentially induced a high production of IFN-γ by CD4^+^ T cells, the skin immunization with iC+CT mainly induced production of IL-17. These results are in accordance with evidence showing the presence of CD4^+^ T cells as producers of IFN-γ in patients, which only control the dissemination of the infection (Fujimura et al., 1996; Koga et al., 2002), while IL-17 production is associated with the optimal clearance of *S. schenckii* and other fungi (Martínez-Álvarez et al., 2014; Ferreira et al., 2015). The high production of IL-17 by CD4^+^ T cells generated with skin immunization could be an effect of the CT as previous reports have mentioned (Tsa et al., 2018). Furthermore this phenotype correlates with the one induced by skin immunization with the CT combined with HEL, as previously reported (Meza-Sánchez et al., 2011).

Another important difference between infection and skin immunization was the production of high levels of IL-2 by skin immunization with iC+CT, compared to the infection with LC. This cytokine is important for T cell proliferation and memory T cell development (Kaech and Wherry, 2007). T_CM_ cells are a population with the capacity to produce high levels of IL-2, and this population was generated by skin immunization with iC+CT, while the infection mainly generated CD4^+^ T cells with an effector phenotype. Previous reports have demonstrated that T cells with an effector phenotype are recruited to the site of infection, while T cells with a circulating memory phenotype (T_CM_ and T_EM_) could have the ability to re-circulate through tissues and lymph nodes (Antia et al., 2005; Zens and Farber, 2015; Stary et al., 2015). These results could explain the increase of CD4^+^ T cells present in the ears of infected mice. Furthermore, it is important to consider the possibility that the infection is not being resolved. Therefore, the presence of LC in the skin could promote a permanent inflammatory environment that preferentially induces the differentiation of CD4^+^ T cells from the sdLN into effector T cells that are recruited to the site of the infection. In this context, in acute viral infection models, it has been demonstrated that the populations of virus specific CD4^+^ and CD8^+^ T cells primed for short periods of time, preferentially develop into memory T cells (Moulton et al., 2006). In addition, in acute viral infection, both central and effector memory phenotypes have been reported (Bingaman et al., 2005). The results are similar to our findings that the immunization of iC+CT could mimic an acute infection. In contrast, during a chronic viral infection, the virus specific CD8 T cells failed to acquire the memory T cell properties (Wherry et al., 2004) which is similar to our results obtained after *S. schenckii* infection.

Nevertheless, despite previous reports mentioning that T cells present in the tissues could mediate a DTH reaction without the participation of the circulating memory T cells (Matheu et al., 2008), our results show that during infection, the T cells present in the skin were not capable of maintaining the DTH after 48 h post-challenge; whereas the DTH induced by skin immunization with the iC+CT that generates circulating memory T cells was stronger and it was maintained even after 48 h post-challenge. These results suggest that T cells present in the skin are important at the beginning of the DTH reaction but the participation of the circulating memory T cells for the maintenance of this response is necessary. Thus, when we depleted circulating CD4^+^ T cells with an anti-CD4 monoclonal antibody without affecting the tissue of resident CD4^+^ T cells, the DTH response was not maintained.

Importantly, the skin immunization that generates an immune response mediated by circulating memory CD4^+^ T cells, which mainly produces IL-17, was translated in a protective immune response mediated by circulating memory (T_CM_ and T_EM_) T cells. In contrast, the infection induced effector non-protective Th1 response that highly expresses TNF-α, which has been implicated in the severity of skin lesions (Romo-Lozano et al., 2012; Alba-Fierro et al., 2016). In addition the infection induce a transitory DTH response could be related with the immune response observed in sporotrichosis patients (Fujimura et al., 1996; Koga et al., 2002). The fact that the IL-17 expressed by skin CD4^+^ T cells is not protective in the infection model could be related to the effector phenotype which contrasts with what is observed in the *Candida albicans* infection mice model that develop an immune response mediated by T cells with a tissue resident memory (T_RM_) phenotype (Park et al., 2017).

Finally, some reports show the capacity of T_RM_ cells to protect against an infection with influenza virus (Hogan et al., 2001; Teijaro et al., 2011; Zens and Farber, 2015) or against other fungal infections such as *Candida albicans* (Park et al., 2017). However, our results show that when the anti-CD4 monoclonal antibody was inoculated into mice, the protection against the infection induced by skin immunization with iC+CT was lost, demonstrating that mainly the circulating memory CD4^+^ T cells mediate protection against the *S. schenckii* infection. These results are in accordance with previous reports showing the importance of the circulating memory T cells generating a protective immune response against infection with *Chlamydia trachomatis* (Matheu et al., 2008; Stary et al., 2015). This is the first report that demonstrates the participation of circulating memory CD4^+^ T cells for the development of a protective immune response against a *S. schenckii* infection.

In sum, there are important differences between the immune response induced by infection and the one induced by skin immunization in mice. We proposed that the reduced induction of circulating (central and effector) CD4^+^ T cell memory response in combination with the presence of effector non-protective CD4^+^ T cells that highly express TNFα is an important mechanism that uses the fungus to induce tissue damage and evade effective immune response and become a chronic infection. This report shows the development and importance of circulating memory CD4^+^ T cells in the clearance of *S. schenckii*, contributing to the current understanding of the development of the memory immune response that could protect against this fungal infection. This could provide some insight into other chronic fungal infections as well as for the design of effective vaccines against this fungus and possibly other chronic infections.

## Author Contributions

LB conceived and directed the project. LB, CT, and AG-L designed the experiments. AG performed the experiments, acquired, and analyzed the data. LA-H was involved in the development of skin experiments. LB, CT, and LA-H contributed reagents, materials, and analysis tools. LB, AG-L, and CT wrote the manuscript. All the authors reviewed the manuscript critically.

## Conflict of Interest Statement

The authors declare that the research was conducted in the absence of any commercial or financial relationships that could be construed as a potential conflict of interest. The reviewer JM-Á and the handling Editor declared their shared affiliation.

## Abbreviations

μg: micrograme
CCR7: chemokine receptor type 7
CD4: cluster of differentiation 4
CD44: cluster of differentiation 44
CD45: cluster of differentiation 45
CD62L: cluster of differentiation 62 Ligand
CD69: cluster of differentiation 69
CT: cholera toxin
DCs: dendritic cells
DTH: delayed-type hypersensitivity
FACS: Fluorescence activated cell sorting
HEL: hen egg lysozyme
i.d.: intradermal
i.p.: intraperitoneal
iC: inactivated conidia
IFN-γ: interferon gamma
IL-17: interleukin 17
IL-2: interleukin 2
IL-4: interleukin 4
Iono: Ionomicine
MFI: Mean Fluorescence Intensity
PBS: phosphate buffered saline
PMA: phorbol 12-myristate 13-acetate
sdLN: skin draining lymph nodes
T_CM_: central memory T cells
TCR-β: T cell receptor beta chain
T_EM_: effector memory T cells
Th1: T helper 1
Th17: T helper 17
T_RM,_: tissue-resident memory T cells
