# Supplementary material for: Sporothrix schenckii Immunization, but Not Infection, Induces Protective Th17 Responses Mediated by Circulating Memory CD4+ T Cells
Source: Front Microbiol. 2018 Jun 12;9:1275. doi: 10.3389/fmicb.2018.01275 (PMC6005866; doi:10.3389/fmicb.2018.01275)
Supplement: Supplementary file 1 [file Data_Sheet_1.PDF]

[redacted]

Supplementary Material

SPOROTHRIX SCHENCKII IMMUNIZATION, BUT NOT INFECTION, INDUCES PROTECTIVE TH17 RESPONSES MEDIATED BY CIRCULATING MEMORY $CD4^+$ T CELLS.

Alberto García-Lozano[1,2], Conchita Toriello[3], Laura Antonio-Herrera[1] and Laura C. Bonifaz[1*].

*** Correspondence:**

Dra. Laura C. Bonifaz

labonifaz@yahoo.com

1.1 Supplementary Figures

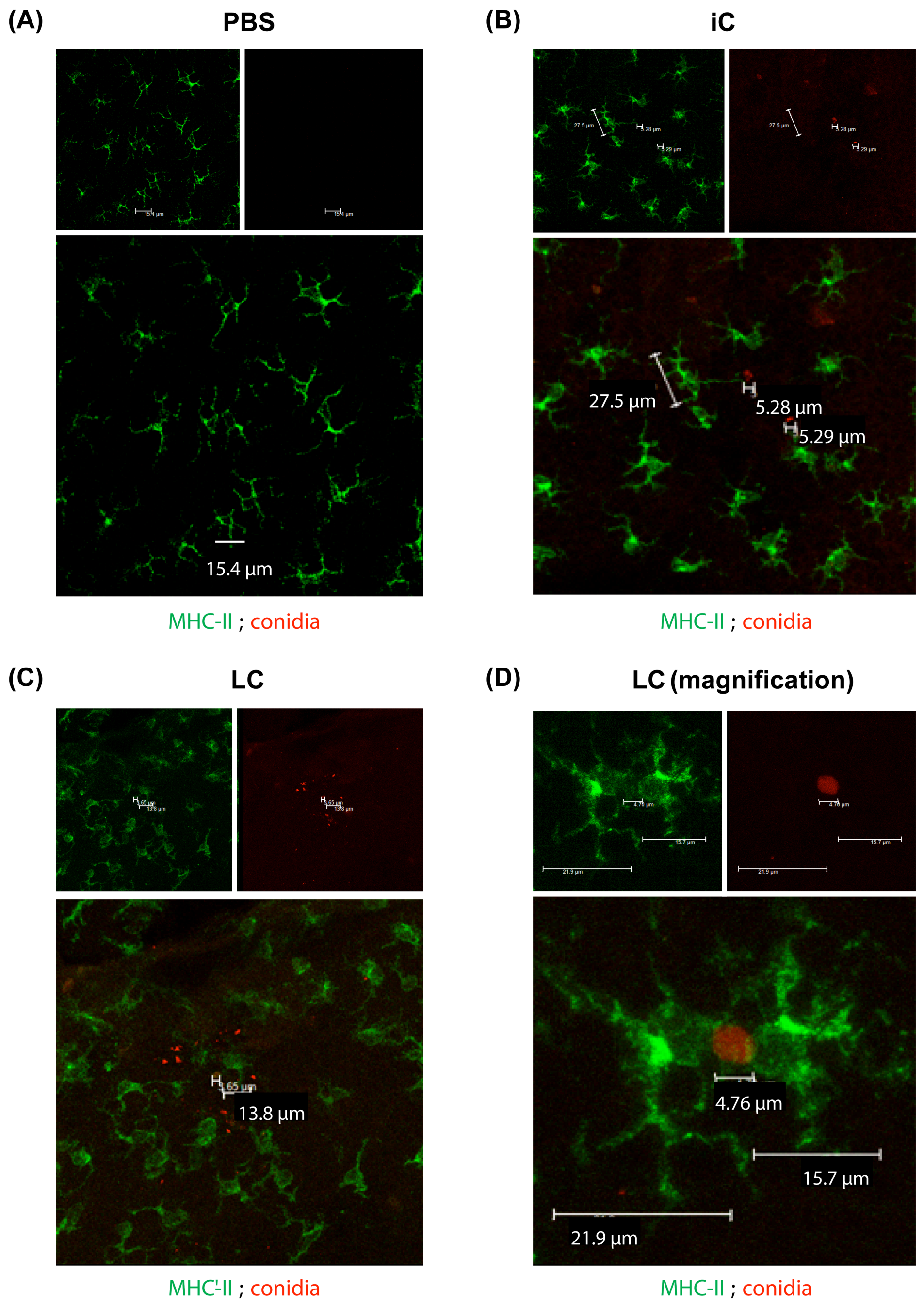

Supplementary Figure 1. Live Conidia of *S. schenckii* preserve the conidia form after the intradermal injection. Transgenic mice C57BL/6 that express the green fluorescent (GFP) under the major histocompatibility complex class II molecule (MHC-II) promoter were i.d. injected in the ears with PBS, iC (1 x 10^6 iC) stained with the CellMask Orange, or LC (1 x 10^6 LC) stained with CellMask Orange. After 6 h, the expression of MHC II-GFP (green fluorescent protein) and the presence of *S. schenckii* conidia (red) in the epidermal layers were analyzed by confocal microscopy. (**A-D**) Representative images show the expression of MHC II-GFP protein and the presence of *S. schenckii* conidia in ear epidermal sheets of mice inoculated with PBS (**A**), iC stained (**B**) or LC stained (**C-D**). The images are representative of three independent experiments. The scale bars show the different dimensions of DC and conidia.

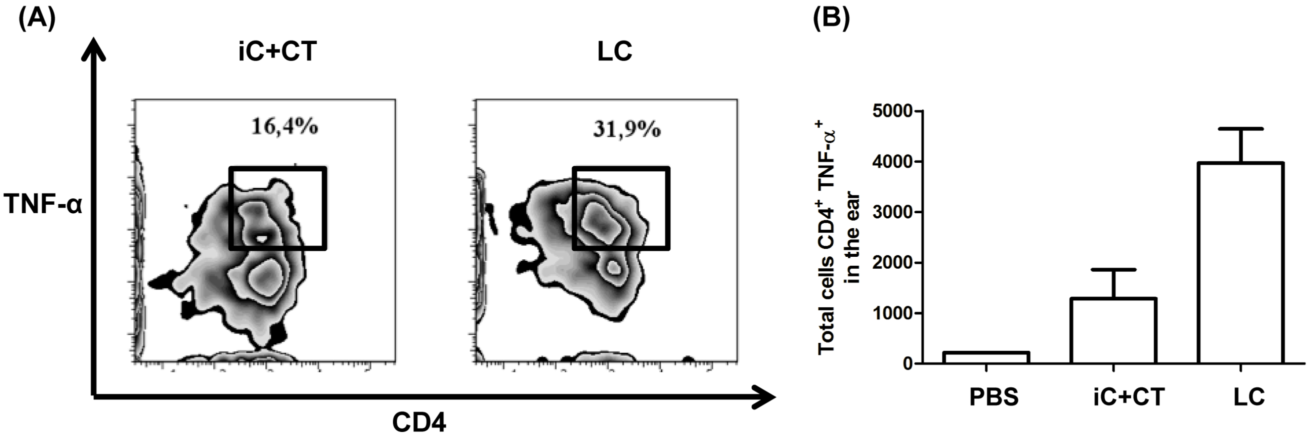

Supplementary Figure 2. Infection with *S. schenckii* induces a high production of TNF-α in the skin. C57BL/6 mice were i.d. injected in the ears with PBS, iC (5 x 10^5 iC) plus the CT (1 µg) (iC+CT) or LC (5 x 10^5 LC). After 14 days, all ear skin cells were obtained and re-stimulated with 5 x 10^5 iC overnight; in the last 4 h of re-stimulation, a cell stimulation cocktail and the protein transport inhibition cocktail were added. These cells were stained for the surface markers CD45, CD4 and TCR-β and for the intracellular cytokine TNF-α. (**A**) Representative plots show the expression of the cytokine TNF-α in the $CD4^+$ T cells. (**B**) The total numbers of $CD45^+$ TCR-β^+ $CD4^+$ TNF-α^+ cells were obtained. The graph shows mean ± SEM data from one independent experiment with four mice per group.
